# Macrophage specific restoration of the *Nrf2* gene in whole-body knockout mice ameliorates steatohepatitis induced by lipopolysaccharide from *Porphyromonas gingivalis* through enhanced hepatic clearance

**DOI:** 10.1371/journal.pone.0291880

**Published:** 2023-10-20

**Authors:** Kanako Chihara, Kosuke Okada, Fumihiko Uchida, Ikuru Miura, Shoichi Komine, Eiji Warabi, Takako Takayama, Hideo Suzuki, Takashi Matsuzaka, Naomi Ishibashi-Kanno, Kenji Yamagata, Toru Yanagawa, Hiroki Bukawa, Junichi Shoda

**Affiliations:** 1 Department of Oral and Maxillofacial Surgery, Institute of Medicine, University of Tsukuba, Tsukuba-shi, Ibaraki, Japan; 2 Doctoral Program in Medical Sciences, Graduate School of Comprehensive Human Sciences, University of Tsukuba, Tsukuba-shi, Ibaraki, Japan; 3 Division of Medical Sciences, Institute of Medicine, University of Tsukuba, Tsukuba-shi, Ibaraki, Japan; 4 Department of Gastroenterology, Institute of Medicine, University of Tsukuba, Tsukuba-shi, Ibaraki, Japan; 5 Faculty of Sports and Health Science, Fukuoka University, Fukuoka-shi, Fukuoka, Japan; 6 Department of Acupuncture and Moxibustion, Faculty of Human Care, Teikyo Heisei University, Toshima-ku, Tokyo, Japan; 7 Division of Biomedical Sciences, Institute of Medicine, University of Tsukuba, Tsukuba-shi, Ibaraki, Japan; 8 Transborder Medical Research Center, University of Tsukuba, Tsukuba-shi, Ibaraki, Japan; Tokyo University of Agriculture, JAPAN

## Abstract

Lipopolysaccharide (LPS) derived from *Porphyromonas gingivalis* (*P*.*g*.), which causes periodontal disease, contributes to the development of non-alcoholic steatohepatitis (NASH). We investigated the role of Nrf2, an antioxidative stress sensor, in macrophages in the development of NASH induced by LPS from *P*.*g*. We generated macrophage-specific *Nrf2* gene rescue mice (*Nrf2*-mRes), which express Nrf2 only in macrophages, using the *cre/loxp* system. Wild-type (WT) mice, whole body *Nrf2*-knockout (*Nrf2*-KO) mice, and *Nrf2*-mRes mice were fed a high-fat diet for 18 weeks, and LPS from *P*.*g*. was administered intraperitoneally for the last 6 weeks. *Nrf2*-KO mice developed severe steatohepatitis with liver inflammation and fibrosis compared with WT mice, and steatohepatitis was ameliorated in *Nrf2*-mRes mice. The mRNA expressions of *Toll-like receptor* (*Tlr*)*-2*, which activates inflammatory signaling pathways after LPS binding, and *α-smooth muscle actin* (*αSma*), which promotes hepatic fibrosis, were reduced in *Nrf2*-mRes mice compared with *Nrf2*-KO mice. The protein levels of LPS-binding protein in livers were increased in *Nrf2*-KO mice compared with WT mice; however, the levels were reduced in *Nrf2*-mRes mice despite similar numbers of F4/80 positive cells, which reflect macrophage/Kupffer cell infiltration into the livers. Nrf2 in macrophages ameliorates NASH through the increased hepatic clearance of LPS.

## Introduction

Non-alcoholic fatty liver disease (NAFLD) is a generic term for the most common chronic liver disease [1, 2], which involves the accumulation of fat in the liver. The number of patients with NAFLD is increasing rapidly alongside the increase in the prevalence of obesity. Non-alcoholic steatohepatitis (NASH), a type of NAFLD, characterized by liver inflammation and fibrosis, is a serious disease that can progress to cirrhosis and hepatocellular carcinoma [3, 4]. The development of NASH is associated with multiple parallel factors, including lipotoxicity, oxidative stress, lipopolysaccharide (LPS) derived from the intestines, and insulin resistance, which form the “multiple parallel hits theory” [5, 6].

Oral biofilm-forming bacteria cause chronic periodontal infection related to bacterial symbiosis disorders that are associated with inadequate oral hygiene [7]. *Porphyromonas gingivalis* (*P*.*g*.) is the main periodontal pathogen, and periodontitis caused by *P*.*g*. is a risk factor for many systemic diseases such as cardiovascular disease, diabetes mellitus, and NAFLD [8]. Recently, odontogenic infection of *P*.*g*. was reported to exacerbate NASH progression, especially liver fibrosis with increased serum LPS [9].

Kupffer cells (KCs), liver resident macrophages that account for approximately 80% of macrophages in the whole body [10], have an important role in innate immunity and the phagocytosis of foreign bodies, including LPS [5]. In NASH subjects, the impaired phagocytic function of KCs decreased the *in vivo* clearance of LPS, and the resulting hyper-endotoxemia accelerated the development of NASH through the upregulation of proinflammatory cytokines induced by LPS [5, 11, 12]. However, exercise training increased the phagocytic ability of KCs for LPS followed by the *in vivo* clearance of exogenously injected LPS [13].

The transcriptional factor nuclear factor E2-related factor-2 (NRF2) is a master regulator of the cellular adaptive response to oxidative stress [14]. Whole-body *Nrf2*-deficient (knockout) (*Nrf2*-KO) mice exhibit a severe deficiency in the gene regulatory program of the antioxidant response, resulting in high susceptibility to oxidative stress-related disease, including NAFLD/NASH [15]. Moreover, Nrf2 was reported to modulate the lipogenic pathway and fatty acid metabolism, and interfere with hepatic lipotoxicity in the NAFLD/NASH model [16, 17]. Furthermore, the activation of NRF2 prevented the LPS-induced upregulation of proinflammatory cytokines, including interleukin (IL)-6 and IL-1β [18]. Previously, the whole-body deletion of Nrf2 impaired the phagocytic function of KCs [19] and immune cells [20]. Furthermore, the overexpression of Nrf2 in myeloid cells unbalanced redox homeostasis, which aggravated DSS-induced acute colitis [21]. Therefore, the role of Nrf2 in macrophages, which have an important role in the immune system of the liver, in NASH is unclear.

The pathophysiological mechanisms involved in the periodontitis-NASH axis in humans are difficult to elucidate because of the complexity of multiple organ communication. Therefore, to investigate these mechanisms, animal models have been developed, including *P*.*g*. dental infection [3], *P*.*g*. oral administration [22], and the intraperitoneal injection of LPS [23].

In the present study, we aimed to clarify the role of Nrf2 in macrophages in LPS metabolism using macrophage-specific *Nrf2* gene rescue mice (*Nrf2*-mRes), in which Nrf2 is only expressed in macrophages. We analyzed NASH pathology induced by injecting LPS from *P*.*g*. into mice fed a high-fat diet (HFD).

## Materials and methods

### Animals

**Fig 1:**
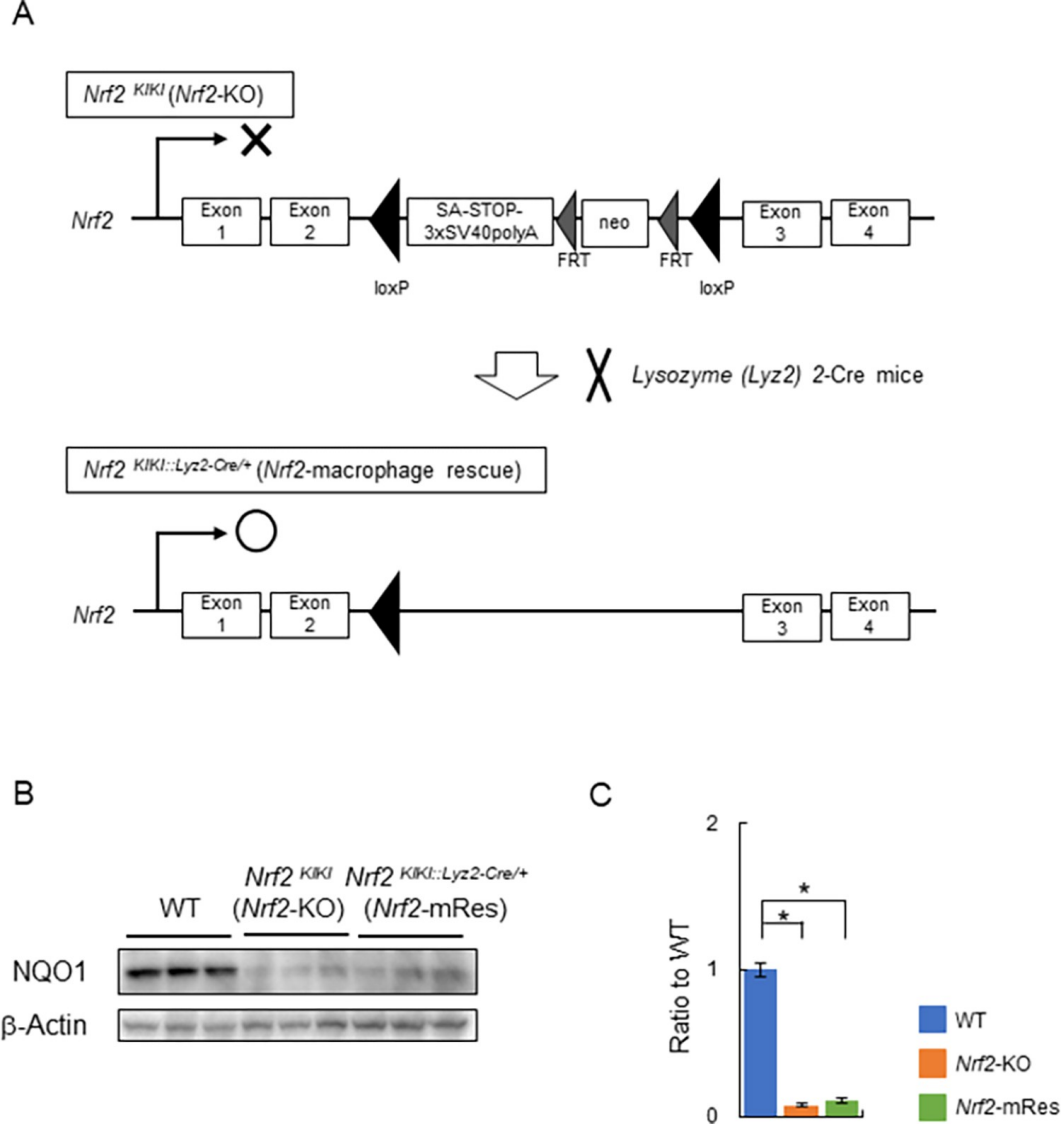
Generation of macrophage-specific *Nrf2* gene rescue mice. (A) Schematic diagram of the design of the *Nrf2* knock-in gene (*Nrf2*^*KIKI*^) and the procedure for generating macrophage-specific *Nrf2* gene rescue mice (*Nrf2*-mRes). *Nrf2*-mRes mice were generated by crossing with Lysozyme 2 (*Lyz2*)-cre mice. (B) Immunoblotting of NAD(P)H:quinone acceptor oxidoreductase 1 (NQO1), a target gene of Nrf2, in liver tissues. (C) The bar graph shows the quantitation of the optical density of the immunoblots.

We generated *Nrf2*-knock-in mice (*Nrf2*^*KIKI*^) on a C57BL/6 background, in which a transcription termination signal flanked by *loxp* sequences and a *polyA-FRT-neomycin resistance gene cassette (neo)-FRT* sequence were inserted into the intron between exons 2 and 3 of the *Nrf2* gene (Fig 1). These mice have whole body *Nrf2* deficiency (*Nrf2*-KO). Macrophage- and KC-specific *Nrf2* gene rescue mice (*Nrf2*^*KIKI*::*Lyz2-Cre/+*^:*Nrf2*-mRes) were obtained by crossing *Nrf2*^*KIKI*^ and Lysozyme (*Lyz2*)-Cre mice using the *cre/loxp* system [24]. The mice were housed in cages at 20–23°C under a 12-h light/dark cycle. Male 5-week-old wild-type mice (WT) (Charles River Laboratories Japan, Kanagawa, Japan), *Nrf2*^*KIKI*^ (*Nrf2*-KO), and *Nrf2*-mRes mice were fed a 60% high-fat/high-sucrose diet (HFD: 60% fat, 24.5% protein, 640 kcal/100 g from Oriental Yeast, Tokyo, Japan) for 18 weeks. In the last 6 weeks of HFD feeding, the mice were administered LPS from *P*.*g*. (0.3 mg/kg body weight, InvivoGen, San Diego, CA) intraperitoneally twice per week. When the mice were 23 weeks of age, liver and blood samples were collected and mice were sacrificed by isoflurane anesthesia. At the end of the experiment, serum and liver tissue specimens were collected for analysis and stored at −80°C. This study was approved by the Animal Care and Use Committee of the University of Tsukuba (approval No. 22–010), and was conducted in accordance with the Regulations on Animal Care and Use in Research, the Welfare and Management of Animals Act, and the Standards for Husbandry, Housing, and Pain Relief of Experimental Animals.

### Intraperitoneal glucose tolerance testing (ipGTT) and insulin (ipITT) tolerance testing

ipGTT was performed at 21 weeks of age and ipITT at 22 weeks of age after 16 hours of fasting. For GTT, a glucose solution (2 mg/g body mass) was administered intraperitoneally and the glucose concentration in tail vein blood was measured for up to 120 minutes. For ITT, insulin (1 mU/g body mass) was administered intraperitoneally and the glucose concentration in tail vein blood was measured for up to 60 minutes. A glucometer (SUGL-001, Shinyo Giken Kogyo Co., Ltd., Niigata, Japan) was used to measure blood glucose concentrations.

### Biochemical analysis

Serum concentrations of aspartate aminotransferase (AST), alanine aminotransferase (ALT), triglyceride (TG), high-density lipoprotein-cholesterol (HDL-CHO), low-density lipoprotein-cholesterol (LDL-CHO), and free fatty acid (FFA) were measured by Oriental Yeast (Tokyo, Japan) using a Hitachi 7180 Auto Analyzer. AST and ALT were measured by the Japan Society of Clinical Chemistry transferable method using an L type Wako AST or ALT-J2 kit (Fujifilm, Tokyo, Japan), TG and FFA were measured by an enzymatic method using L type Wako TG-M or NEFA HF (Fujifilm), and HDL or LDL-CHO were measured by a direct method using CHOLESTEST N HDL or LDL (Sekisui Medical, Tokyo Japan). Glutathione (GSH) and glutathione disulfide (GSSG) levels in the liver tissue were measured using a GSSG/GSH Quantification Kit (Fujifilm).

### Histological analysis

Liver tissue specimens were fixed in 4% paraformaldehyde, embedded in paraffin, and stained with hematoxylin–eosin (HE) and Sirius Red solution using standard protocols. To determine the histopathological severity of steatohepatitis, steatosis, activity, and fibrosis (SAF) scores were assessed by a specialist as follows: steatosis grade (0–3, quantities of lipid droplets), activity (0–4, lobular inflammation and hepatocyte ballooning), and stage of fibrosis (0–4, fiber extension) [25]. An all-in-one fluorescence microscope (BZ-X800, Keyence, Osaka, Japan) was used for imaging. The percentages of sections that were Sirius red positive were calculated using BZ-H4C analysis software (Keyence).

### TG and FFA concentrations in liver tissues

TG and FFA concentrations in the liver tissue specimens were measured using reagent kits (Fujifilm).

### Real-time quantitative polymerase chain reaction (qPCR)

mRNA levels in the liver tissues were determined by real-time quantitative PCR. Total RNA was extracted from liver specimens and cDNA was synthesized using a PrimeScript RT reagent kit (Takara Bio, Shiga, Japan). qPCR was performed with Fast SYBR Green Master Mix (Thermo Fisher Scientific, Waltham, MA, USA) using a BioRad C1000 Touch Thermal Cycler (BioRad). Data were normalized to the amounts of glyceraldehyde 3-phosphate dehydrogenase (*Gapdh*) present and cyclophilin A present in each sample and then averaged. The primers used for qPCR are shown in Table 1.

**Table 1:** Primers used for real-time quantitative PCR.

Gene	Forward primer (5´ - 3´)	Reverse primer (5´ - 3´)
*Ppar α*	TGGGGATGAAGAGGGCTGAG	GGGGACTGCCGTTGTCTGT
*Ppar γ*	TGTCGGTTTCAGAAGTGCCTTG	TTCAGCTGGTCGATATCACTGGAG
*Srebp-1c*	CGGCGCGGAAGCTGT	AGTCACTGTCTTGGTTGTTGATGAG
*Fas*	ATCCTGGAACGAGAACACGATCT	AGAGACGTGTCACTCCTGGACTT
*Tnfα*	AAGCCTGTAGCCCACGTCGTA	GGCACCACTAGTTGGTTGTCTTG
*IL-1β*	TCCAGGATGAGGACATGAGCAC	GAACGTCACACACCAGCAGGTTA
*Tlr-2*	CCCTTCTCCTGTTGATCTTGCT	CGCCCACATCATTCTCAGGTA
*Tlr-4*	GCAGCAGGTGGAATTGTATCG	TGTGCCTCCCCAGAGGATT
*Tlr-6*	AACCTTACTCATGTCCCCAAAGAC	GCATCCGAAGCTCAGATATAGAGTT
*Tlr-9*	CCTTGACAACCTCCCCAAGA	AGGACTTCCAGGTTGGGTAGGA
*αSma*	ACCAACTGGGACGACATGGAA	TGTCAGCAGTGTCGGATGCTC
*Tgf-β1*	GTGTGGAGCAACATGTGGAACTCTA	TTGGTTCAGCCACTGCCGTA
*Col1a1*	GCACGAGTCACACCGGAACT	AAGGGAGCCACATCGATGAT

### Immunoblot analysis

Liver tissue specimens were homogenized in RIPA buffer (Fujifilm Wako Pure Chemical Corporation, Osaka, Japan) and the total protein concentration of each sample was measured by a BCA protein assay kit (Thermo Fisher Scientific). Protein lysate was mixed with Laemmli sample loading buffer (BioRad, CA, USA), and equal amounts of protein were separated by SDS-PAGE. The isolated proteins were transferred to PVDF membranes (BioRad), which were blocked using Blocking One (Nacalai Tesque, Kyoto, Japan). Antibodies were obtained as follows: anti-NAD(P)H:quinone acceptor oxidoreductase 1 (NQO1) from Abcam (ab2346, Cambridge, UK), anti-lipopolysaccharide binding protein (LBP) from Proteintech (23559-1-AP, Tokyo, Japana), anti-F4/80 from Hycult Biotech (HM1066, Uden, Yhe Netherlamds,), anti-hexanoyl-lysine (HEL) from JaiCA, (MHL-021P, Shizuoka, Japnan), and anti-β-actin from Sigma (A5441, Deisenhofen, Germany). Target proteins were visualized using Chemi-Lumi One Super (Nacalai Tesque), and were quantified using ImageJ software (NIH, MD, USA).

### Statistics

Statistical analysis was conducted using IBM SPSS Statistics 26.0 (IBM, Armonk, NY, USA). Values are given as the mean ± standard error of the mean (SEM). Differences were evaluated using a two-tailed unpaired Student’s *t*-test when two groups were compared. Comparisons between multiple groups were performed by one-way ANOVA, followed by Tukey’s multiple comparison test. *P* < 0.05 was considered to represent statistical significance.

## Results

### Nrf2 expression in macrophages does not affect body mass, glucose tolerance, or insulin resistance

**Fig 2:**
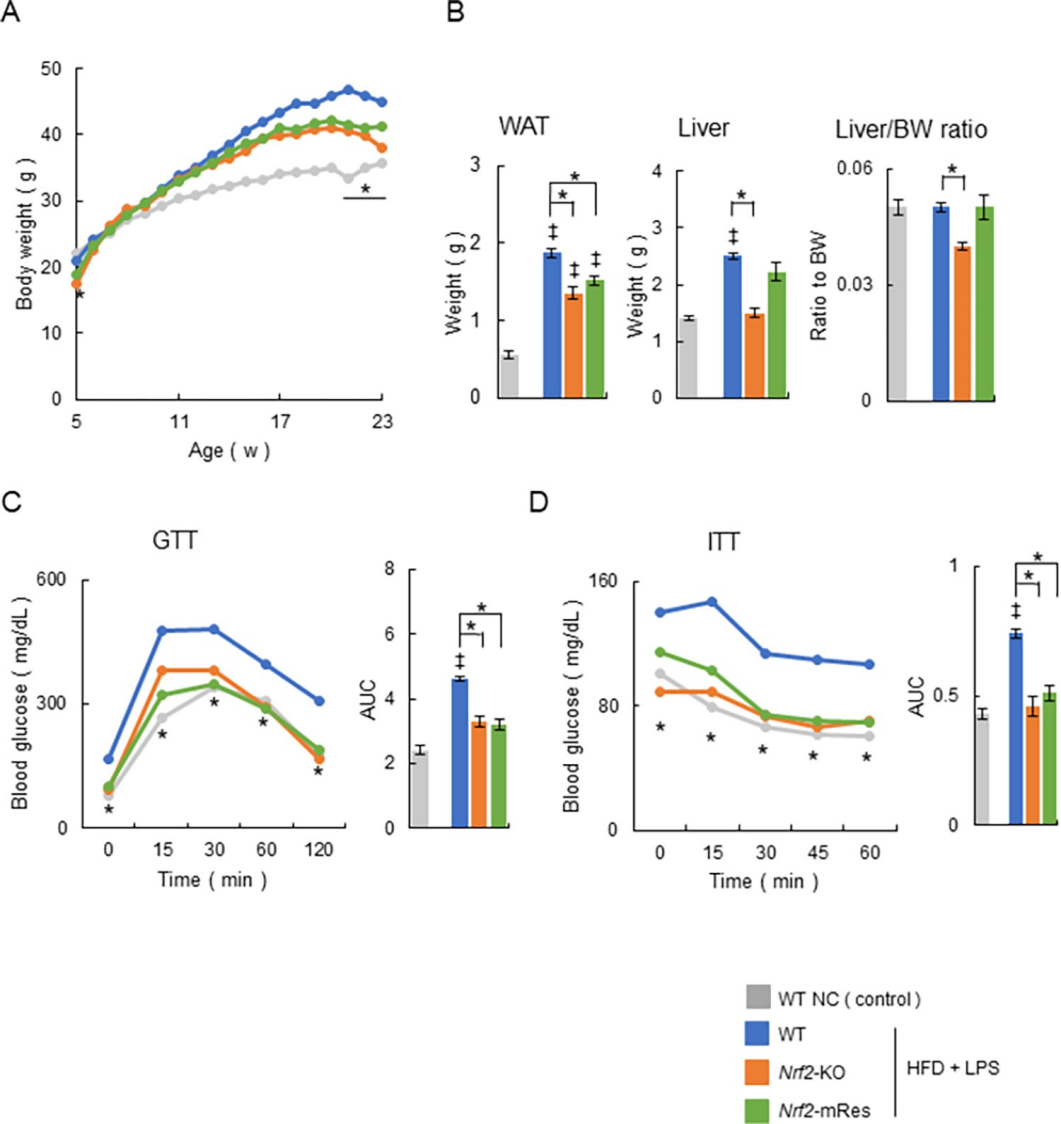
*Nrf2* gene rescue in macrophages does not affect body mass and insulin tolerance. (A) Time course of changes in the body mass of wild-type (WT) mice fed normal chow (NC), and WT, *Nrf2-*KO, and *Nrf2*-mRes mice fed a high-fat diet (HFD) and administrated lipopolysaccharide (LPS) from *Porphyromonas gingivalis* (*P*.*g*.) (WT NC; n = 7, WT HFD + LPS; n = 14, *Nrf2-*KO HFD + LPS; *n* = 9, *Nrf2*-mRes HFD + LPS; *n* = 14). (B) Weights of white adipose tissues around the testis (WAT), liver, and liver weight ratio to body weight (BW) in each group at 23 weeks of age (n = 7–14/group). (C) Changes in blood glucose concentration and area under the curve (AUC) during intraperitoneal glucose tolerance testing (ipGTT) in mice at 21 weeks of age (*n* = 7–14/group). (D) Changes in blood glucose concentration and AUC during intraperitoneal insulin tolerance testing (ipITT) in mice at 22 weeks of age (*n* = 7–14/group) mice. Values are the mean ± SEM. ‡*P* < 0.05, WT NC vs. HFD + LPS groups, *P < 0.05, WT HFD + LPS vs. *Nrf2-*KO HFD + LPS or *Nrf2*-mRes HFD + LPS.

The body weights of WT mice fed normal chow (NC) (control group), or WT, *Nrf2*-KO, and *Nrf2*-mRes mice fed an HFD were monitored from 5 to 23 weeks of age. At birth, the body weights of *Nrf2*-KO and *Nrf2*-mRes mice were slightly lower than those of WT mice (WT; 20.9 ± 0.1 g, *Nrf2*-KO; 17.5 ± 0.4 g, *Nrf2*-mRes; 18.8 ± 0.4 g, Fig 2A). The body weights of mice fed an HFD were significantly higher than those of WT controls after 11 weeks of age. After 20 weeks of age, *Nrf2*-KO and *Nrf2*-mRes mice stopped gaining weight and their body weights were similar at 23 weeks of age; however, the body weights of *Nrf2*-KO mice were significantly lower than those of WT mice (WT; 44.9 ± 0.4 g, *Nrf2*-KO; 38.0 ± 1.0 g, *Nrf2*-mRes; 41.3 ± 1.1 g, Fig 2A). The weights of white adipose tissue around the testis increased significantly in mice fed an HFD compared with the WT NC group. The weights of white adipose tissue, livers, and the liver/body weight ratio were lower in *Nrf2*-KO and *Nrf2*-mRes mice compared with WT mice, which had similar body weight changes; however, there were no differences between the *Nrf2*-KO and *Nrf2*-mRes mice (Fig 2B). These results indicate that Nrf2 in macrophages does not affect the body mass associated with *Nrf2* gene deletion.

The glucose tolerance and insulin resistance of WT, *Nrf2*-KO, and *Nrf2*-mRes mice were evaluated using ipGTT and ipITT. ipGTT in *Nrf2*-KO and *Nrf2*-mRes mice was characterized by lower blood glucose concentrations following glucose administration and a smaller area under the glucose curve compared with WT mice fed an HFD, which was increased compared with the WT NC group; however, there was no difference between the *Nrf2*-KO and *Nrf2*-mRes mice (Fig 2C). Similarly, ipITT in *Nrf2*-KO and *Nrf2*-mRes mice showed lower fasting and post-insulin blood glucose concentrations, and a smaller area under the curve compared with WT mice fed an HFD, which was increased compared with the WT NC group (Fig 2D). These results indicate that Nrf2 in macrophages does not affect glucose tolerance and insulin resistance.

### Nrf2 expression in macrophages ameliorates hepatic inflammation and fibrosis

**Fig 3:**
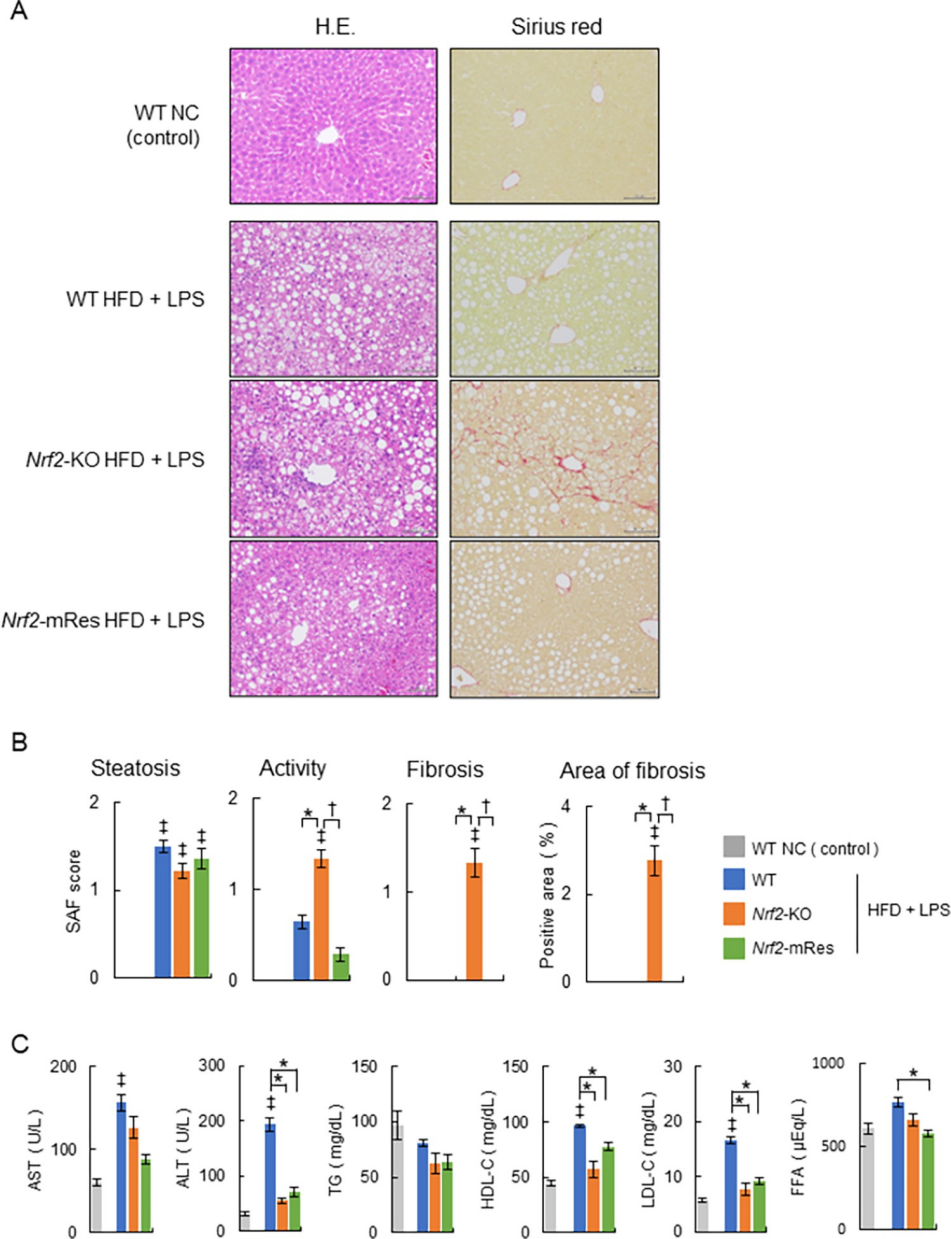
Nrf2 expression in macrophages ameliorates the progression of NASH. (A) Representative hematoxylin and eosin (HE) and Sirius red-stained sections of livers from 23-week-old wild-type (WT), *Nrf2-*KO, and *Nrf2*-mRes mice fed a normal chow (NC), or an HFD and administrated lipopolysaccharide (LPS) from *Porphyromonas gingivalis* (*P*.*g*.). Scale bars represent 100 μm. (B) Steatosis, activity, fibrosis (SAF) scores, and areas positive for Sirius red staining (*n* = 7–14/group). (C) Serum biochemistry of aspartate aminotransferase (AST), alanine aminotransferase (ALT), triglyceride (TG), high-density lipoprotein-cholesterol (HDL-CHO), low-density lipoprotein-cholesterol (LDL-CHO), and free fatty acid (FFA) (*n* = 5–9/group). Values are the mean ± SEM. ‡*P* < 0.05, WT NC vs. HFD + LPS groups, *P < 0.05, WT vs. *Nrf2-*KO or *Nrf2*-mRes in HFD + LPS groups, ^†^*P* < 0.05, *Nrf2-*KO vs. *Nrf2*-mRes in HFD + LPS groups.

The liver histology of WT, *Nrf2*-KO, and *Nrf2*-mRes mice is shown in Fig 3A. To determine the histopathological severity of steatohepatitis, SAF scores were assessed (Fig 3B). HFD feeding and LPS administration induced steatohepatitis in all three groups of mice, namely fat droplet deposition and inflammatory cell infiltration. Moreover, steatosis histology and SAF scores were similar between all groups. Hepatic inflammation and fibrosis in *Nrf2*-KO mice were more severe compared with WT mice; however, histology and SAF scores were significantly ameliorated in *Nrf2*-mRes mice compared with *Nrf2*-KO mice (WT vs *Nrf2*-KO vs *Nrf2*-mRes: activity: 0.6 ± 0.1 g vs 1.3 ± 0.1 g vs 0.3 ± 0.1 g; fibrosis: 0.0 ± 0.0 g vs 1.3 ± 0.2 g vs 0.0 ± 0.0 g, Fig 3A and 3B). Similarly, the area of Sirius red staining was larger in *Nrf2*-KO mice compared with WT mice; however, that of *Nrf2*-mRes mice was ameliorated compared with *Nrf2*-KO mice (WT vs *Nrf2*-KO vs *Nrf2*-mRes: area of Sirius red staining: 0.01 ± 0.00% vs 2.77 ± 0.35% vs 0.02 ± 0.00%, Fig 3B). These findings imply that Nrf2 in macrophages ameliorates hepatic inflammation and fibrosis.

A comparison of blood biochemistry after liver injury showed AST levels in *Nrf2*-KO and *Nrf2*-mRes mice tended to be lower compared with those in WT HFD + LPS mice, which were increased compared with those in WT NC mice. In addition, ALT levels in *Nrf2*-KO and *Nrf2*-mRes mice were lower than those in WT HFD + LPS mice, which were increased compared with those in WT NC mice; however, there was no difference in levels between *Nrf2*-KO and *Nrf2*-mRes mice (Fig 3C). The inconsistency between inflammation observed by histology and liver injury assessed from serum may be attributable to a difference in the time course of the peak of liver injury, which was indicated by serum AST and ALT levels. The serum lipid profiles of TG, HDL-CHO, LDL-CHO, and FFA were assessed (Fig 3C, right panels). TG in the HFD + LPS groups tended to be lower compared with that in the WT NC group; however, there was no difference in levels between the WT, *Nrf2*-KO, and *Nrf2*-mRes HFD + LPS mice. HDL-CHO and LDL-CHO levels in the WT HFD + LPS group were increased compared with the WT NC group and were higher than those in *Nrf2*-KO and *Nrf2*-mRes HFD + LPS mice; however, there was no difference between *Nrf2*-KO and *Nrf2*-mRes mice. FFA in the WT HFD + LPS group was higher than that in *Nrf2*-mRes HFD + LPS mice; however, there was no difference between *Nrf2*-KO and *Nrf2*-mRes mice.

### Triglyceride and fatty acids in steatohepatitis livers

**Fig 4:**
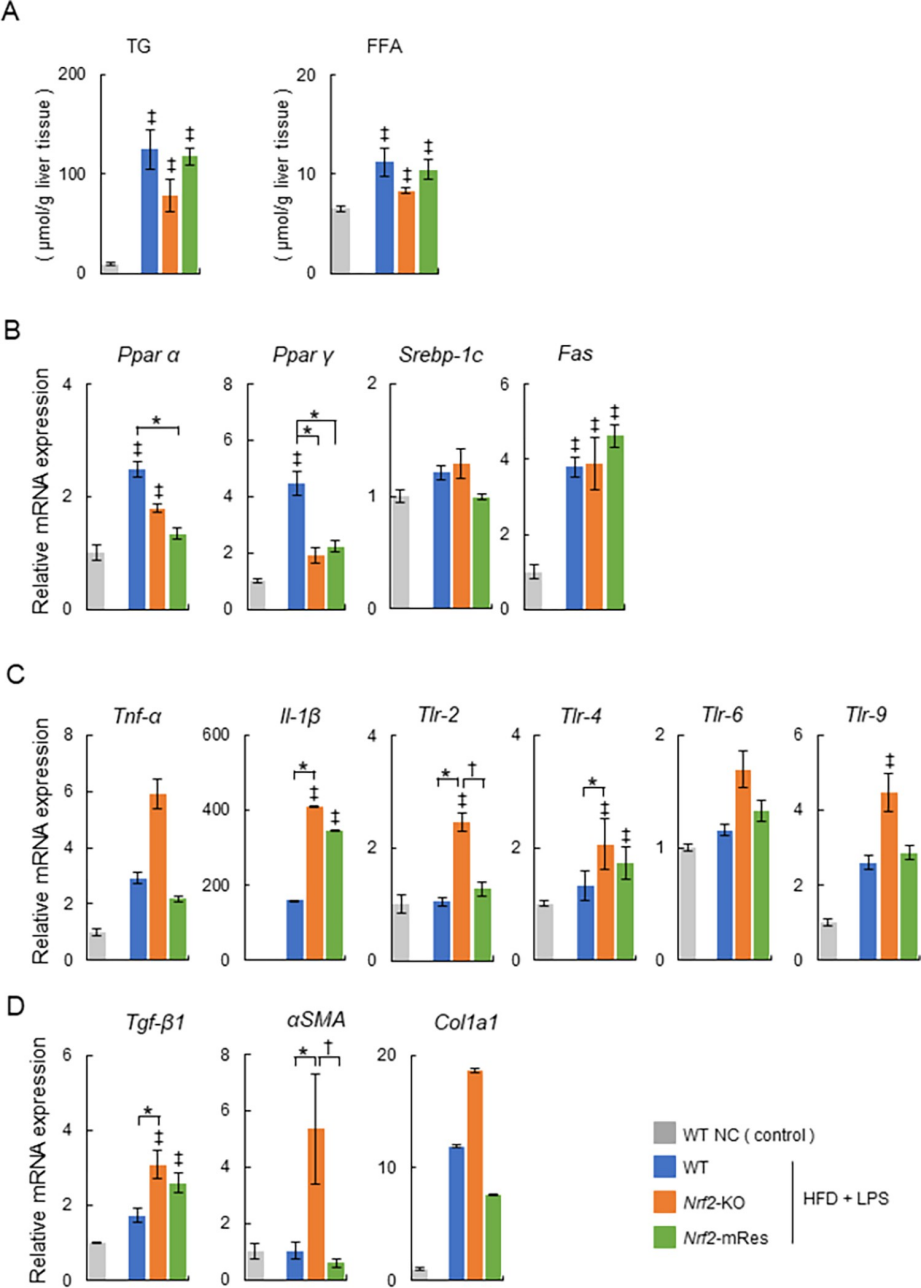
Analysis of triglyceride (TG) and free fatty acids (FFA) in livers, and lipid metabolism, inflammatory signaling genes, Toll-like receptors, and fibrosis-related genes in the livers of wild-type (WT), *Nrf2-*KO, and *Nrf2*-mRes mice fed normal chow, or an HFD and administrated lipopolysaccharide (LPS) from *Porphyromonas gingivalis* (*P*.*g*.). (A) TG and FFA in the liver tissues. (B) mRNA levels of factors related to lipid and fatty acid metabolism (*Ppar α*, *Ppar γ*, *Srebp-1c*, and *Fas*, *n* = 5–9/group). (C) mRNA levels of inflammatory cytokines (*Tnf-α* and *Il-1β*) and Toll-like receptors (*Tlr*)-2, -*4*, -*6*, and -*9* (*n* = 5–9/group). (D) mRNA levels of fibrosis-related genes (*Tgf-β1*, *αSma*, and *Col1a1*, *n* = 5–9/group). Values are the mean ± SEM. ‡*P* < 0.05, WT NC vs. HFD + LPS groups, *P < 0.05, WT vs. *Nrf2-*KO or *Nrf2*-mRes in HFD + LPS groups, ^†^*P* < 0.05, *Nrf2-*KO vs. *Nrf2*-mRes in HFD + LPS groups.

The effects of HFD + LPS on lipid profiles in liver tissues are shown in Fig 4A. TG and FFA contents in the livers of mice in the HFD + LPS group were increased compared with those in the WT NC group, which correlated with steatosis demonstrated by the histological analysis of livers (Fig 3A and 3B). Furthermore, in the HFD + LPS group, the TG and FFA contents in *Nrf2*-KO mice tended to be decreased compared with those in WT *Nrf2*-mRes mice; however, there was no significant difference between WT, *Nrf2*-KO, and *Nrf2*-mRes mice.

Hepatic mRNA expression levels related to lipid and fatty acid metabolism in the liver tissues were measured by qPCR (Fig 4B). The mRNA levels of *peroxisome proliferators activated receptor (Ppar) α* and *Ppar γ*, which are fatty acid oxidases, were increased in the WT HFD +LPS group compared with the WT NC group. These trends were observed in the *Nrf2*-mRes HFD + LPS group; however, there was no significant difference between *Nrf2*-KO and *Nrf2*-mRes mice (Fig 4B). We measured the mRNA levels of *sterol regulatory element-binding protein (Srebp)-1c* and *fatty acid synthase (Fas)*, factors involved in fatty acid *de novo* synthesis. The mRNA levels of *Fas* were higher in the HFD + LPS groups than in the WT NC group, and this trend was similar for *Srebp-1c*; however, there was no difference in the levels of *Fas* and *Srebp-1c* between *Nrf2*-KO and *Nrf2*-mRes mice (Fig 4B). These results indicate that Nrf2 in macrophages does not affect hepatic lipid and fatty acid metabolism in our mouse model.

### Nrf2 expression in macrophages attenuates signaling related to hepatic inflammation and fibrosis

Hepatic mRNA expression levels of inflammatory cytokines (*Tnf-α* and *Il-1β*), *Toll-like receptors* (*Tlr-2*, *-4*, *-6*, and -*9*, Fig 4C), and fibrosis-related genes (*Tgf-β1*, *αSma*, and *Col1a1*, Fig 4D) were measured by qPCR. Many of these factors were increased in the HFD + LPS groups compared with the WT NC group. The mRNA levels of *Tlr-2*, which activates inflammatory signaling by LPS, and *αSma*, were increased in *Nrf2*-KO HFD + LPS mice compared with WT HFD + LPS mice, and significantly attenuated in *Nrf2*-mRes HFD + LPS mice compared with *Nrf2*-KO HFD + LPS mice. Similarly, the mRNA levels of *Il-1β*, *Tlr-4*, and *Tgf-β1* were increased in *Nrf2*-KO HFD + LPS mice compared with WT HFD + LPS mice, and tended to be attenuated in *Nrf2*-mRes HFD + LPS mice compared with *Nrf2*-KO HFD + LPS mice. The mRNA levels of *Tnf-α*, *Tlr-6*, *-9*, and *Col1a1* tended to be increased in *Nrf2*-KO HFD + LPS mice compared with WT HFD + LPS mice, and tended to be attenuated in *Nrf2*-mRes HFD + LPS mice compared with *Nrf2*-KO HFD + LPS mice (Fig 4C and 4D). These findings suggest that Nrf2 in macrophages inhibits signaling associated with hepatic inflammation and fibrosis, which are activated through LPS and TLR.

### Nrf2 expression in macrophages recovers LPS clearance and reduces oxidative stress in the liver

**Fig 5:**
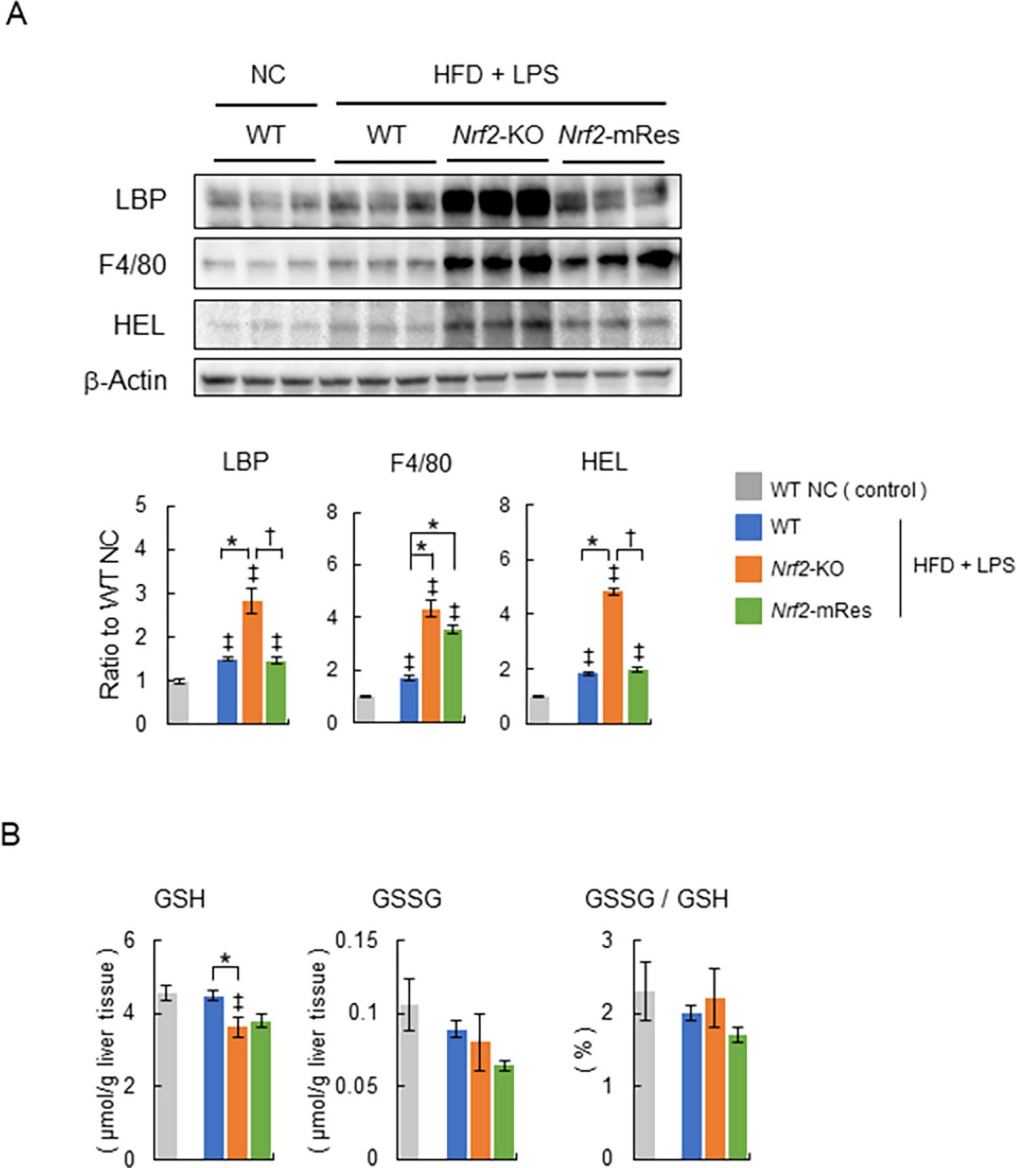
The expression of Nrf2 in macrophages recovers the clearance of LPS and does not affect the infiltration of Kupffer cells in a mouse model of NASH. (A) Representative immunoblots of LPS-binding protein (LBP), F4/80, hexanoyl lysine (HEL), and β-actin in liver tissues from wild-type (WT), *Nrf2-*KO, and *Nrf2*-mRes mice fed normal chow, or an HFD and administered lipopolysaccharide (LPS) from *Porphyromonas gingivalis* (*P*.*g*.) (*n* = 5–6/group). Bar graphs in the right panel show the quantitation of the optical density of the immunoblots. (B) Glutathione (GSH), glutathione disulfide (GSSG) concentrations, and the percentage of GSSG/GSH in the livers of WT, *Nrf2-*KO, and *Nrf2*-mRes mice fed normal chow, or an HFD and administered LPS from *P*.*g*. (*n* = 5–6/group). Values are the mean ± SEM. ‡*P* < 0.05, WT NC vs. HFD + LPS groups, *P < 0.05, WT vs. *Nrf2-*KO or *Nrf2*-mRes in HFD + LPS groups, ^†^*P* < 0.05, *Nrf2-*KO vs. *Nrf2*-mRes in HFD + LPS groups.

Immunoblot analyses of LBP, which reflects LPS in the liver tissues, F4/80, which indicates the infiltration of macrophages into livers (i.e. KC infiltration), and HEL, which is an oxidative stress marker of early lipid peroxidation, were performed to investigate the molecular mechanism related to how Nrf2 in macrophages protects against hepatic inflammation and fibrosis. These proteins were significantly increased in the HFD + LPS group compared with the NC group (Fig 5A). The protein levels of LBP were increased in *Nrf2*-KO HFD + LPS mice compared with WT HFD + LPS mice; however, these increases were decreased in *Nrf2*-mRes HFD + LPS mice. The protein levels of F4/80 were increased in *Nrf2*-KO HFD + LPS and *Nrf2*-mRes HFD + LPS mice compared with WT HFD + LPS mice (Fig 5A). These results indicate that the expression of Nrf2 in macrophages can recover the clearance of LPS and does not affect the infiltration of KCs in a mouse model of NASH.

The protein levels of HEL were increased in *Nrf2*-KO HFD + LPS mice compared with WT HFD + LPS mice; however, these increases in HEL as well as LBP were decreased in *Nrf2*-mRes HFD + LPS mice (Fig 5A). Levels of GSH, which is consumed when removing reactive oxygen species from the liver, were decreased significantly in *Nrf2*-KO HFD + LPS mice compared WT HFD + LPS mice (Fig 5B). Levels of GSSG and the percentage of GSSG/GSH, which accumulates by oxidative stress, were similar between the three HFD +LPS groups (Fig 5B). Because Nrf2 was deleted in the hepatocytes of *Nrf2*-KO and *Nrf2*-mRes mice, these changes suggest that the increased oxidative stress in the livers was a result of hepatic inflammation caused by an overload of LPS, which was altered by the deletion of Nrf2 in macrophages.

## Discussion

In the present study, we demonstrated that Nrf2 in macrophages had a protective role against the development of NASH in *Nrf2*-mRes mice induced by an HFD and *P*.*g*.-derived LPS. This might be explained by the importance of Nrf2 in macrophages related to phagocytic activity and increasing the clearance of LPS from the liver.

Determining the role of Nrf2 in the periodontitis-LPS-NASH axis is complicated because the individual characteristics of Nrf2 in macrophages are difficult to analyze using whole-body Nrf2 deleted mice. To clarify the role of Nrf2, we generated *Nrf2*-mRes mice, which express the *Nrf2* gene only in macrophages, derived from *Nrf2*-knock-in mice (*Nrf2*^*KIKI*^, Fig 1). Using these mice, the influence of *Nrf2* deletion in other tissues on accumulated visceral fat, myokines in muscles, intestine permeability influenced by barrier function, and/or the microbiota can be eliminated. Interestingly, the macrophage-specific expression of Nrf2 ameliorated hepatic inflammation and fibrosis compared with the whole-body deletion of Nrf2, similar to the WT mice in the present study (Fig 3A and 3B). Moreover, the infiltration of KCs into the liver, demonstrated by immunoblotting of F4/80, was similar in *Nrf2*-KO and *Nrf2*-mRes mice; however, the levels of LBP, which reflects LPS levels in the liver, were increased in *Nrf2*-KO mice compared with *Nrf2*-mRes mice (Fig 5A). Although a direct LPS clearance experiment was not assessed in this study, these results indicate that the rescue of Nrf2 in macrophages recovered the clearance of LPS from the liver, decreased the hepatic expression of *Tlr-2*, and tended to decrease genes encoding the proinflammatory cytokines *Tnf*-*a* and *Il*-*1β* (Fig 4A). These findings suggest that Nrf2 in macrophages from the liver (KCs) reduced the LPS-induced inflammatory response in these mice. Additionally, the expression of *αSma*, which induces hepatic fibrosis, was also reduced by the expression of Nrf2 in macrophages (Fig 4B). LPS derived from *P*.*g*. was reported to activate hepatic stellate cells through TLRs, TGF-β1, and Smad [26], and the results of the present study suggest that the reduction of LPS by Nrf2 expression in macrophages downregulated hepatic stellate cell activation and suppressed hepatic fibrosis in *Nrf2*-mRes mice. The decreased body weight of *Nrf2*-KO and *Nrf2*-mRes mice might be related to decreased muscle mitochondrial function [27]; however, body weight, glucose tolerance, and insulin resistance were not affected by Nrf2 rescue in macrophages (Fig 2A, 2C and 2D). Moreover, Nrf2 in macrophages did not affect hepatic lipid and fatty acid metabolism in our mouse model. These results suggest that the amelioration of NASH-associated inflammation and fibrosis may occur secondary to the restoration of LPS clearance induced by the rescue of Nrf2 independent of insulin resistance.

The mechanisms involved in the periodontitis-LPS-NASH axis in humans are difficult to analyze because the complicated cascade involves multiple factors and organs [1, 5, 7]. In the present study, we used an intraperitoneal injection of LPS from *P*.*g*. with HFD as a model of NASH with periodontitis to focus on the role of Nrf2 in macrophages in LPS metabolism. This model has some limitations. The delivery of LPS directly to the liver is not physiological, and therefore it does not recapitulate periodontitis. Moreover, alterations in the gut microbiota by periodontal pathogenic bacteria are difficult to analyze.

In human studies, the phagocytic capacity of immune cells was reported to influence the development of NASH. A reduction in the phagocytic capacity of KCs was restored by long-term high-intensity exercise, which was followed by the amelioration of inflammation and oxidative stress in NAFLD patients [28]. Moreover, an improvement in periodontitis after exercise and oral hygiene instructions was reported to ameliorate NAFLD in humans [29, 30]. Therefore, the control of periodontitis and LPS derived from periodontal disease-related bacteria such as *P*.*g*., may be a promising new option for the management of NASH.

Recently, two strong genetic risk factors for fatty liver diseases, including NAFLD, were identified as missense variants in patatin phospholipase-like domain containing protein 3 (PNPLA3) [31] and in transmembrane 6 superfamily member 2 (TM6SF2), which promotes bulk lipidation and prevent fatty liver disease [32]. However, whether genetic abnormalities of Nrf2 affect the onset and progression of NAFLD and NASH in humans has not been reported, and there was no difference in lipid profiles between *Nrf2*-KO and *Nrf2*-mRes mice (Figs 3C, 4A and 4B).

In summary, we showed that Nrf2 in macrophages had a protective effect on the development of NASH induced by an HFD and *P*.*g*.-derived LPS. The activation of Nrf2 in macrophages may be a new option for the management of NAFLD associated with periodontal diseases.

## Supporting information

S1 Raw images The uncut original western blotting images data. They are whole and original images because they were cut the membranes according to size of each target before probing by primary antibody using the size marker (Precsion Plus Protein Kaleidoscope, Bio-Rad).
(PDF)

## Abbreviations

ALT: alanine aminotransferase
AST: aspartate aminotransferase
αSMA: α smooth muscle actin
Col1a1: collagen 1a1
Fas: fatty acid synthase
GSH: glutathione
GSSG: glutathione disulfide
GTT: glucose tolerance test
HEL: hexanoyl lysine
IL-1β: interleukin-1β
ITT: insulin tolerance test
KO: knockout
LBP: LPS-binding protein
LPS: lipopolysaccharide
Lyz2: lysozyme 2
NASH: non-alcoholic steatohepatitis
NRF2: nuclear factor-E2-related factor-2
P.g.: *Porphyromonas gingivalis*
Ppar: peroxisome proliferators activated receptor
Srebp-1c: sterol regulatory element-binding protein-1c
TGF-β1: transforming growth factor-β1
TLR: Toll-like receptor
TNF-α: tumor necrosis factor-α
WAT: white adipose tissue
WT: wild-type
