# Supplementary material for: Macrophage specific restoration of the Nrf2 gene in whole-body knockout mice ameliorates steatohepatitis induced by lipopolysaccharide from Porphyromonas gingivalis through enhanced hepatic clearance
Source: PLoS One. 2023 Oct 20;18(10):e0291880. doi: 10.1371/journal.pone.0291880 (PMC10588835; doi:10.1371/journal.pone.0291880)
Supplement: S1 Raw images — They are whole and original images because they were cut the membranes according to size of each target before probing by primary antibody using the size marker (Precsion Plus Protein Kaleidoscope, Bio-Rad). (PDF) [file pone.0291880.s001.pdf]

Supplement figure

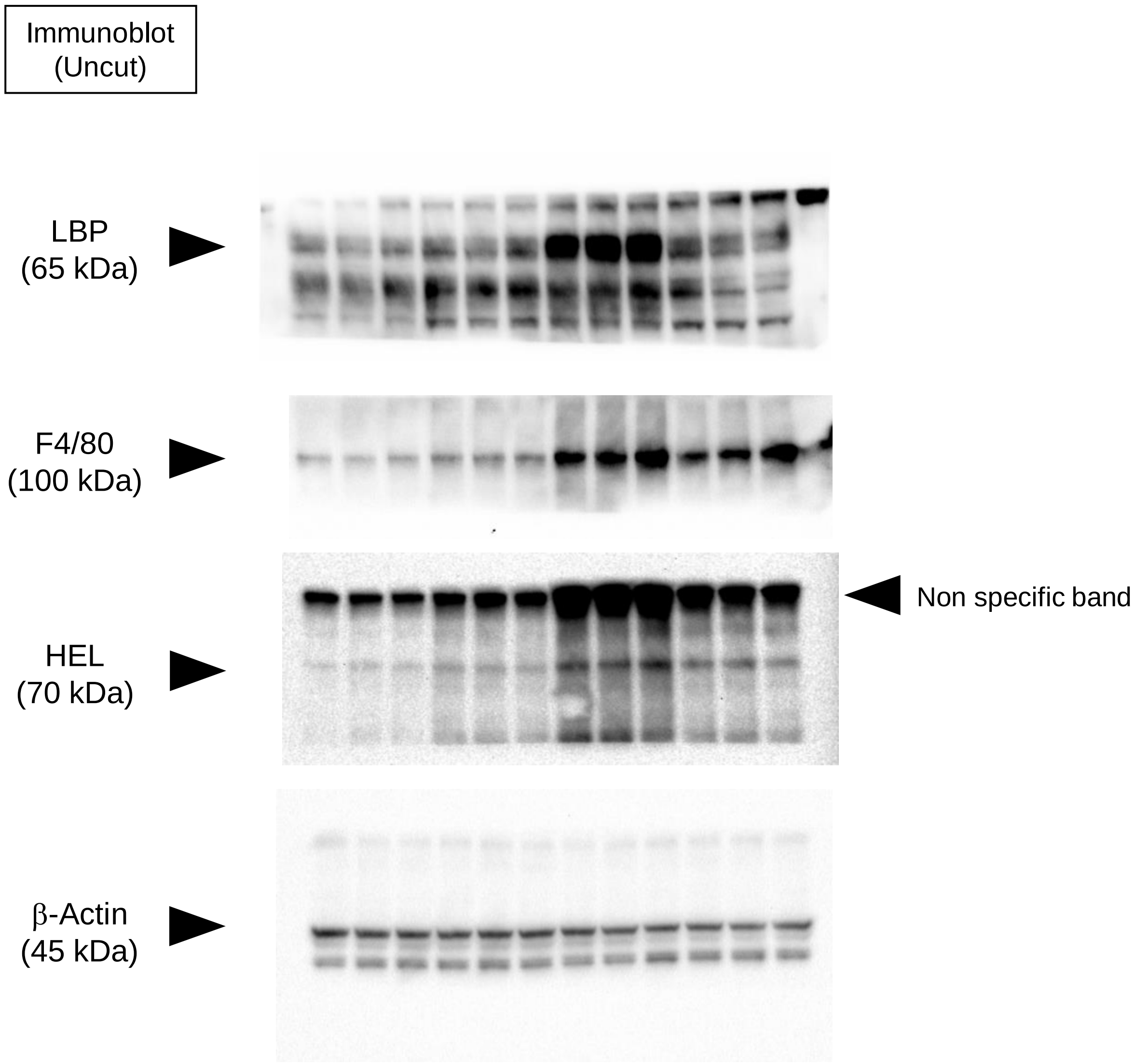

Supplement figure:
The uncut original Western Blotting images data. They are whole and original images because they were cut the membranes according to size of each targets before probing by primary antibody using the size marker (Precsion Plus Protein Kaleidoscope, Bio-Rad). So we cannot see the bands of size marker in these images because they were provided in the membranes.
